# *In vivo* efficacy of combination therapy with albendazole and atovaquone against primary hydatid cysts in mice

**DOI:** 10.1007/s10096-021-04230-5

**Published:** 2021-03-26

**Authors:** Shigehiro Enkai, Hirokazu Kouguchi, Daniel Ken Inaoka, Takao Irie, Kinpei Yagi, Kiyoshi Kita

**Affiliations:** 1 Department of Pediatrics, Teikyo University School of Medicine, 2-11-1 Kaga, Itabashi-ku, Tokyo, 173-8605 Japan; 2 School of Tropical Medicine and Global Health, Nagasaki University, 1-12-4 Sakamoto, Nagasaki, 852-8523 Japan; 3 Department of Infectious Diseases, Hokkaido Institute of Public Health, N19 W12, Kita-Ku, Sapporo, Hokkaido 060-0819 Japan; 4 Department of Molecular Infection Dynamics, Shionogi Global Infectious Diseases Division, Institute of Tropical Medicine (NEKKEN), Nagasaki University, Nagasaki, 852-8523 Japan; 5 Laboratory of Veterinary Parasitic Diseases, Department of Veterinary Sciences, Faculty of Agriculture, University of Miyazaki, 1-1 Gakuen-Kibanadai Nishi, Miyazaki, 889-2192 Japan; 6 Department of Host-Defense Biochemistry, Institute of Tropical Medicine (NEKKEN), Nagasaki University, 1-12-4 Sakamoto, Nagasaki, 852-8523 Japan

**Keywords:** Alveolar echinococcosis, *Echinococcus multilocularis*, Albendazole, Atovaquone, Mitochondrial complex III, Combined administration, Combination therapy, Drug development, Synergy

## Abstract

Alveolar echinococcosis (AE) is caused by the larval stage of *Echinococcus multilocularis*. Chemotherapy for AE involves albendazole (ABZ), which has shown insufficient efficacy. More effective chemotherapy for AE is needed. Previously, we have demonstrated that atovaquone (ATV), an antimalarial, inhibits mitochondrial complex III of *E*. *multilocularis* and restricts the development of larval cysts in *in vivo* experiments. Therefore, in this study, we evaluated the efficacy of ABZ and ATV combination therapy on *E*. *multilocularis* in culture and *in vivo* experiments. Protoscoleces were treated with 50 μM ABZ and/or ATV in the medium; the duration of parasite elimination was determined under aerobic and anaerobic culture. In the *in vivo* experiment, the effects of ABZ and ATV combination treatment in BALB/c mice infected orally with eggs from the feces of an adult-stage *E. multilocularis*-infected dog were compared with those of standard oral ABZ therapy. In the culture assay, the duration of elimination associated with ABZ and ATV combination treatment was shorter than that associated with ATV alone under aerobic conditions. Protoscolex viability progressively reduced owing to the combination treatment under anaerobic conditions; however, either drug used singly did not exhibit antiparasitic effects under hypoxia. Furthermore, compared with ABZ alone, the combination treatment significantly reduced the growth of the primary cyst in the liver of mice infected orally with parasite eggs (*P* = .011). ATV enhances the effect of ABZ in the treatment of AE in mice.

## Introduction

Alveolar echinococcosis (AE) is a helminthic zoonotic disease caused by infection with the larval stage of the cestode parasite *Echinococcus multilocularis* [1]. The prevalence of AE is highest in China, Central Asia, Russia, parts of Europe, and Japan [1]. Adult-stage *E. multilocularis* release their eggs into the feces of the definitive host. The accidental ingestion of the eggs by intermediate hosts, such as small rodents, leads to the release of infective larvae (oncospheres) in the intestinal lumen. The oncospheres mainly reach the liver *via* the circulatory system, in which they grow into metacestodes (larval cysts) containing protoscoleces. After predation of the infected rodent by Canidae such as foxes, the protoscoleces transform and develop into adult tapeworms in the small intestine. Humans are infected as an aberrant intermediate host upon accidentally swallowing the parasite eggs. The growth of larval cysts in the liver leads to life-threatening conditions, such as organ dysfunction, several years after infection.

Radical surgical resection of the parasitic mass is the basis of treatment for AE and is usually accompanied by chemotherapy [1, 2]. In cases with inoperable advanced cysts of AE, the chemotherapeutic treatment involves albendazole (ABZ), which is not adequately efficacious [3]. During the clinical course, chemotherapy plays an important role in the process of treatment as an increase in the number of larval cysts in the liver is associated with a poor prognosis. Hence, many *in vivo* experiments have been performed to assess the efficacy of combination therapy with ABZ and other compounds [4].

We focused on the mitochondrial respiratory systems of the parasite as the drug target in this study. *E. multilocularis* employs an aerobic respiratory pathway, oxidative phosphorylation, or an anaerobic pathway, fumarate respiration, for its survival in various oxygen conditions [5–7]. The eggs of *E. multilocularis* excreted from the definitive host are exposed to oxygen in the long term. The oncosphere and metacestodes (larval cysts) reside in the artery and liver of the intermediate host, respectively. These stages, which are the target of treatment, can access the portal vein and hepatic vein that exhibit high arterial oxygen tension. However, it is difficult for these stages to access oxygen owing to their size and site of infection. Adult worms live in the intestine of the definitive host, under low-oxygen conditions. A similar change in the respiratory system is also found in other parasites [8–11].

Briefly, oxidative phosphorylation generally involves complexes I to IV and V. Complex I oxidizes NADH and passes electrons to ubiquinone. Complex II receives electrons from succinate and functions as succinate-quinone reductase. Ubiquinone transfers the reducing equivalent to complex III, which passes it to complex IV via cytochrome *c*. Proton-motive force is generated across the inner mitochondrial membrane at complexes I, III, and IV, which drive ATP synthesis through complex V. In contrast, fumarate respiration involves complexes I, II, and rhodoquinone. Complex I acts as a proton pump that is driven by the oxidation of NADH and contributes to the reduction of rhodoquinone. Complex II accepts electrons from rhodoquinol and catalyzes the reduction of fumarate to succinate as a terminal oxidase. Quinol-fumarate reductase activity is the reverse reaction of the succinate-quinone reductase activity of complex II under anaerobic conditions. As the advantage of fumarate respiration, ATP can be synthesized by the proton-pump activity of complex I and ATP synthase (complex V) even under low-oxygen conditions.

We have previously reported that atovaquone (ATV), a cytochrome *bc*_1_ complex inhibitor and antimalarial agent, inhibits mitochondrial complex III of *E*. *multilocularis* at extremely low concentrations and decreases the development of larval cysts in *in vivo* experiments [5]. However, in the case of *E*. *multilocularis*, the effect of ATV alone is limited compared with that of ABZ in treated mice because ATV inhibits only aerobic respiration [5]. Therefore, in this study, the *in vivo* effects of combination therapy with ABZ and ATV were compared to those of standard oral ABZ treatments in mice with primary hydatid cysts after being orally infected with eggs of *E*. *multilocularis* as a natural infection model.

## Methods

### Culture assay of live *E. multilocularis* protoscoleces

A culture assay was performed to determine the efficacy of the combination of ABZ and ATV. The protoscoleces obtained were cultured in Connaught Medical Research Laboratories 1066 medium (Gibco, Grand Island, NY, USA) containing 0.5% (w/v) yeast extract (Difco Laboratories, Detroit, MI, USA), 23 mM 4-(2-hydroxyethyl)-1-piperazineethanesulfonic acid, 0.5% (w/v) D (+)-glucose, 0.4 mM sodium taurocholate (Wako Pure Chemical Industries, Osaka, Japan), 57 mM sodium hydrogen carbonate, 2 mM L-glutamine (Gibco), 100 U/mL penicillin, and 100 μg/mL streptomycin (Gibco). Half of the medium was changed on day 3. For anaerobic cultures, six-well plates were sealed in plastic containers with oxygen-detecting agents and oxygen scavengers (Aneromeito®, Nissui Pharmaceutical, Tokyo, Japan) to maintain the oxygen concentration under 0.3% at 37 °C. The protoscoleces were treated with ABZ (Tokyo Chemical Industry, Tokyo, Japan) and/or ATV (Tokyo Chemical Industry) at a final concentration of 50 μM in the culture medium, and the duration of parasite elimination was determined. The control group was supplemented with 0.5% (v/v) dimethyl sulfoxide (DMSO), and all conditions were assayed in triplicate. The viability of protoscoleces was determined by microscopic observation of more than 170 protoscoleces per well using the trypan blue exclusion test.

### *In vivo* studies to determine the efficacy of ABZ and ATV combination treatment in BALB/c mice with primary hydatid cysts

The *in vivo* effects of ABZ and ATV combination treatment were evaluated and compared with those of standard oral ABZ treatments. BALB/c mice (female, body weight of 25 g, 9 weeks old, and daily average food intake of 4.0 g) were infected orally with 100 μL of a suspension containing 200 eggs obtained from the feces of an adult *E. multilocularis*-infected dog in biosafety level 3. Three mice were euthanized to confirm the presence of cysts (diameter, 1–2 mm; length, 1–2 mm) in the liver 4 weeks after infection (Fig. 2a). After confirming the growth of larval cysts, 33 BALB/c mice were divided into the following 4 groups: 6 mice in the ABZ group, 9 mice in the ABZ and ATV combination treatment group, 9 mice without treatment, and 9 mice in the ATV group. The no-treatment and ATV groups were considered as the control group to assess whether this *in vivo* experiment was performed appropriately compared with that reported previously. ABZ and ATV were mixed into the feed at doses of 125 mg/100 g and 133 mg/100 g, respectively, as reported previously [5]. Each agent was blended with pulverized feed by using a Waring blender until a uniform consistency was achieved [12]. Water was added to the clayish feed, and pellets (2 × 2 × 3 cm) were prepared using a spatula. The treatments were performed for 8 weeks by feeding the mice with the drug-mixed feed ad libitum from 8 weeks after infection. The mice were euthanized to investigate the 8-week therapeutic effect. Necropsies were performed, and the proportion of cysts on the liver surface was determined using digital image analysis software (ImageJ, Bethesda, MD, USA) to evaluate the development of larval cysts.

### Statistical analysis

The culture assays were performed in triplicate, and the results were each expressed as the mean ± standard error of the mean (SEM) calculated using Microsoft Office Excel 2016 (Microsoft Corporation, Redmond, WA, USA). The results of the *in vivo* experiments were analyzed using one-way analysis of variance and a multiple comparison test. *P* < .05 was considered significant. All statistical analyses were performed using EZR (version R3.6.3; Saitama Medical Center, Jichi Medical University, Saitama, Japan) [13].

## Results

**Fig. 1:**
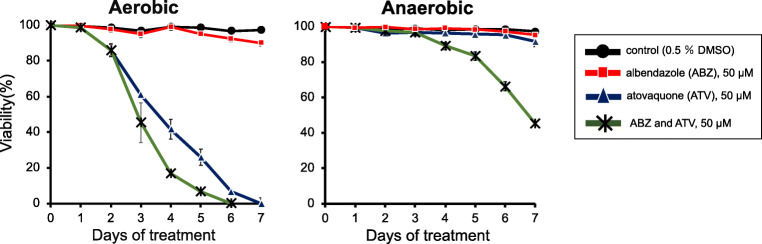
Culture assay under aerobic and anaerobic conditions (O_2_ < 0.3%). *Echinococcus multilocularis* protoscoleces were treated with albendazole (ABZ), atovaquone (ATV), and ABZ plus ATV at a final concentration of 50 μM in each culture medium. One control group was supplemented with 0.5% (v/v) dimethyl sulfoxide alone. The protoscoleces were observed daily for seven consecutive days, and their viability was assessed by performing the trypan blue exclusion test. The results represent the means and standard deviation values of at least three samples

The results of the culture assay under aerobic and anaerobic conditions are shown in Fig. 1. The protoscoleces in the DMSO control group maintained 98 to 100% viability throughout the experimental period under both conditions. In the culture assay under aerobic conditions, ATV treatment eliminated *E. multilocularis* protoscoleces completely by day 7. ABZ killed only 10% of the protoscoleces by day 7. The viability of the protoscoleces progressively reduced during *in vitro* treatment with ABZ plus ATV compared with ATV alone. In the culture assay under anaerobic conditions, ABZ or ATV did not kill any protoscolex by day 7. However, ABZ plus ATV resulted in the elimination of 55% of the protoscoleces by day 7 in the hypoxic culture.

**Fig. 2:**
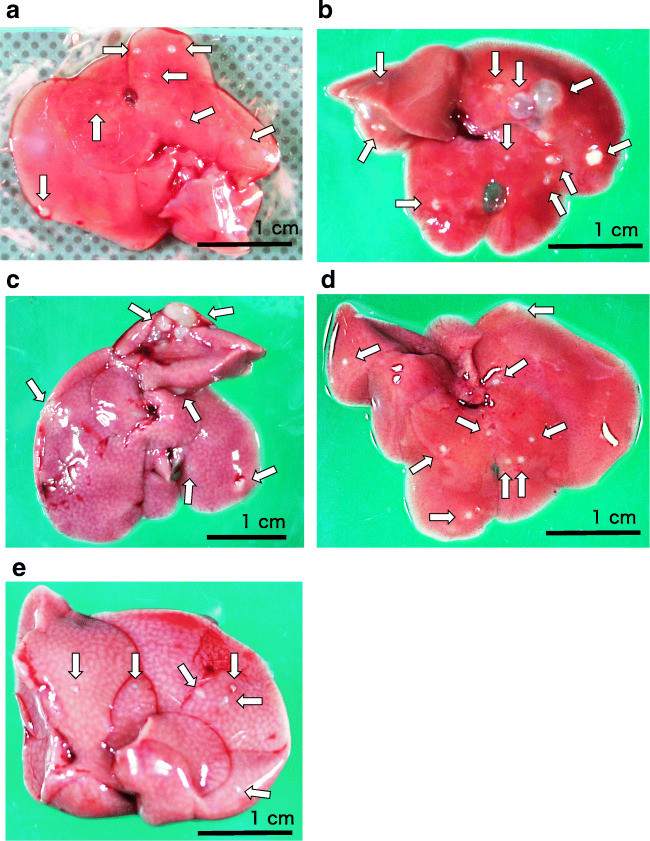
Liver lesions in mice (BALB/c) infected orally with a suspension containing 200 eggs obtained from the feces of a dog infected with adult *E. multilocularis*. Representative samples are selected around large cysts. Before treatment (**a**): cysts on the liver 4 weeks after infection. No treatment (**b**): the condition of the liver of an untreated mouse 16 weeks after oral infection with parasite eggs. Atovaquone (ATV) (**c**), albendazole (ABZ) (**d**), and ABZ plus ATV (**e**): all groups were orally administered with each agent for 8 weeks from 8 weeks after infection. The whitish dots and cystic masses on the livers represent infection with established parasites. The proportion of the cyst area on the liver was determined in detail with the digital image analysis software. The proportion of cysts on the liver surface was **a** 0.73%, **b** 6.5%, **c** 4.0%, **d** 0.65%, and **e** 0.29%

**Fig. 3:**
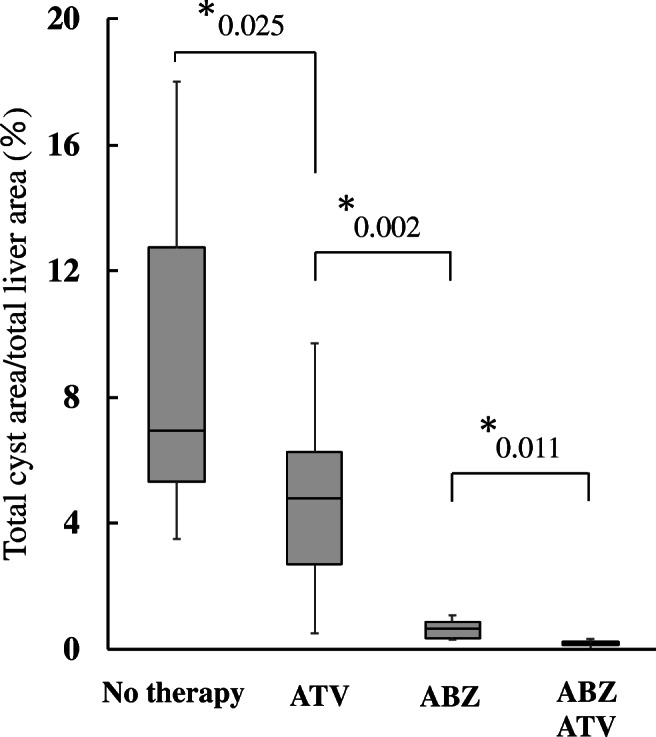
*In vivo* effects of albendazole (ABZ) and atovaquone (ATV) combination therapy on primary alveolar hydatid cysts in a mouse model. The cyst area on the liver in the ABZ/ATV treatment group was compared with that in the group treated with standard oral ABZ treatment. No-treatment and ATV groups were created to assess whether this *in vivo* experiment was performed appropriately compared with that reported previously [6]. The proportion of cysts on the surface of the liver was measured by digital image analysis. *P-value of <.05 at 95% confidence level was considered significant

Figure 2 shows representative liver conditions around large cysts before and after treatment to allow a better understanding of the primary hydatid cysts in the mouse liver. Larval cysts, 0.04 to 0.09 cm in diameter, were disseminated on the surface of the liver 4 weeks after infection and before treatment (Fig. 3a). Larval cysts of various shapes and sizes were observed, with a maximum size of 0.3 cm 16 weeks after the oral administration of eggs in the untreated mouse liver (Fig. 3b). The cysts treated by ATV had irregular shapes and various sizes on the liver (Fig. 3c). After ABZ treatment, cysts with uniform shape and diameter of 0.05 to 0.1 cm were observed on the liver (Fig. 3d). In the samples treated by the combination of ABZ and ATV, cysts had a diameter of 0.03 to 0.1 cm and a slightly irregular margin (Fig. 3e). The proportion of cysts on the surface of the liver in Fig. 3a–e was 0.74, 6.5, 4.0, 0.65, and 0.29%, respectively.

A significant difference in the area of the larval cysts was observed between the no-treatment and ATV groups (*P* = .025). Compared with ABZ alone, combination treatment with ABZ and ATV significantly reduced the formation of cysts in the mouse liver (*P* = .011; Fig. 3).

## Discussion

In this study, we assessed the viability of protoscoleces under normoxic and hypoxic culture conditions upon treatment with ABZ and/or ATV. ATV eliminated the parasites strongly under aerobic conditions but not under anaerobic conditions because the target of ATV is complex III, which is not a component in the anaerobic respiratory chain (fumarate respiration) [5]. ABZ only eliminated less than 10% of the protoscoleces under aerobic and anaerobic conditions, although it inhibited the growth of cysts *in vivo.* It is not clear how the elimination speed in ABZ culture changes after day 8 in this study. The metabolites of ABZ and the immune system of the host might contribute to the effects of ABZ *in vivo*, although the mechanism of action of *ABZ* involves the inhibition of tubulin polymerization in the intestinal cells of the parasite [14]. Moreover, the duration of elimination associated with the combined administration of ABZ and ATV was 1 day shorter than that associated with ATV alone under aerobic conditions. Notably, the viability of protoscoleces progressively decreased during the culture assay of the parasite with the coadministration of ABZ and ATV under anaerobic conditions, although the administration of either agent alone did not result in any antiparasitic effect. The result of culture assay supports the significant reduction of the cyst area by the *in vivo* combination treatment.

An *in vivo* experiment was performed to evaluate the efficacy of ABZ and ATV combination treatment against primary hydatid cysts in infected mice. We demonstrated that the area of the cysts *in vivo* was significantly restricted by the combination of ABZ and ATV. Under aerobic conditions, ABZ permeation might be enhanced by cell membrane damage due to reactive oxygen species (ROS) generation caused by ATV in cyst areas, from the aerobic respiratory chain, considering that complexes I, II, and III of the electron transport chain are reported to be the major sites of ROS production [15, 16]. The oxidation of either complex I or II substrates in the presence of complex III inhibitors increases the production of ROS [17, 18]. Damage to the respiratory chain by the excessive production of ROS leads to electron transfer dysfunction, which causes irreversible cell disorder. It is assumed that cellular dysfunction attributed to ROS contributed to the potentiating effect of ABZ under aerobic conditions. Additionally, under hypoxia, the efficacy of the treatment *in vivo* may be enhanced by the synergistic action of ABZ and ATV, as shown in the anaerobic culture assay. ATV inhibits not only complex III but also dihydroorotate dehydrogenase (DHODH), which has been annotated in the genome data of *E. multilocularis* [19] and other parasites [20, 21]. DHODH contributes to adaptation to anaerobiosis by regulating fumarate reductase activity and pyrimidine synthesis [22]. The dysfunction of anaerobic metabolism pathways may be synergistically induced by the inhibition of DHODH and the action of ABZ. However, little is known about the importance of DHODH in the biological functions of *E. multilocularis*.

Here, we focused on the culture assay and *in vivo* experiments. However, our study has limitations. First, the biochemical approach was not utilized to demonstrate the synergistic action and hypothesis mentioned above. Second, morphological and pathological changes were not observed through electron microscopy and tissue section of cysts before and after treatment. Further studies will demonstrate the role of ATV and ABZ in the enhancement of potency in the aerobic and anaerobic metabolic pathway.

In conclusion, compared with ABZ alone, combined treatment with ABZ and ATV reduced the area of larval cysts in mouse liver significantly. This finding can provide ideas for further study of the treatment of and drug development for *E. multilocularis* infection.

## Data Availability

Not applicable
